# Genetic diversity and novel haplotypes of *Apis mellifera jemenitica* on the Arabian Peninsula: insights from mtDNA markers

**DOI:** 10.3389/fgene.2025.1532988

**Published:** 2025-04-25

**Authors:** Mohammed Alsharhi, Ahmad Al-Ghamdi, Maged Ahmed Al-Garadi, Mohamed Alburaki

**Affiliations:** ^1^ Agriculture Department, College of Agriculture and Veterinary Medicine, Thamar University, Dhamar, Yemen; ^2^ Chair of Engineer Abdullah Bugshan for Bee Research, Plant Protection Department, College of Food and Agriculture Sciences, King Saud University, Riyadh, Saudi Arabia; ^3^ Animal Production Department, College of Food and Agriculture Sciences, King Saud University, Riyadh, Saudi Arabia; ^4^ United States Department of Agriculture Agricultural Research Service (USDA-ARS), Bee Research Laboratory, Beltsville, MD, United States

**Keywords:** *Apis mellifera* jemenitica, genetic diversity, mtDNA, cytochrome b, COI-COII haplotype, COI gene

## Abstract

The genetic diversity of *Apis mellifera jemenitica* populations collected from the Arabian Peninsula (Saudi Arabia, Yemen, and Oman), Jordan, and Ethiopia, was examined using three mtDNA markers: 1- Cytochrome *b* (Cyt *b*), 2- Cytochrome *c* oxidase I (COI) and 3- The intergenic region located between the cytochrome *c* oxidase I & II (COI-COII). DNA was extracted from 44 samples, amplified for each region using classic PCR, and the resulting amplicons were sequenced using Sanger technology at both ends. Sequences were verified and aligned, and Maximum-Likelihood phylogenetic analyses were conducted with reference sequences from other subspecies. The *in silico* DraI mtDNA COI-COII (DmCC) test was applied to the COI-COII sequences to identify evolutionary lineages and haplotypes. Moreover, COI-COII haplotype network analyses were conducted to assess the intra- and inter-genetic relationships between samples and references. Based on the Cyt *b* marker, most samples cluster within the African lineage (A) near *lamarckii* and *syriaca* (Sub-lineage Z) subspecies. Few samples from Ethiopia and Yemen were closely related to *simensis* and *scutellata* clades. The COI gene separated *jemenitica* samples (Bootstrap = 97) from subspecies of other lineages (C and O). The DmCC test revealed a P0Q2 structure in the intergenic region for all samples, with a distinct 18 bp deletion in the P0 element observed in two Ethiopian and one Yemeni samples, suggesting *litorea* or *simensis* origin. A total of 13 COI-COII haplotypes were identified, among which 8 haplotypes were novel: Saudi Arabia (1), Yemen (3), Oman (1), and Ethiopia (3), with a haplotype diversity (*H*) of 0.980. Furthermore, molecular-variance parsimony in COI-COII confirmed a distant genetic relationship between Ethiopian samples versus samples of the Arabian Peninsula. The haplotype network analysis suggests a higher intra-*jemenitica* diversity than previously understood with a *syriaca* ancestry to this clade. These findings offer crucial insights into the conservation of *A*. *m*. *jemenitica* and its role in preserving biodiversity in arid ecosystems. Additionally, the data enhance our understanding of the genetic diversity of *A. m. jemenitica* and its evolutionary connections with other neighboring African subspecies.

## 1 Introduction

*Apis mellifera jemenitica* Ruttner (1976) is a major honey bee subspecies of the African evolutionary lineage (A). In his book “Biogeography and Taxonomy of Honeybees,” Ruttner discussed this poorly studied subspecies under the Chapter “Honeybees of Tropical Africa” alongside a handful of other African subspecies such as *lamarckii*, *litorea*, *scutellata*, *adansonii*, *monticola*, *capensis* and *unicolor* (Ruttner, 1988). Based on morphometrical characteristics, *jemenitica* is considered one of the most intriguing and significant subspecies of *A. mellifera*, notable for its extreme morphology and ecology. It was not recognized as a taxonomic unit until 1976 (Ruttner, 1988). This subspecies extends into a large, hot, arid territory of 4,500 km from East to West, covering many countries, including Yemen, Saudi Arabia, Oman, Ethiopia, Chad, Somalia, and Sudan (Dutton et al., 1981; Ruttner, 1988; Ruttner et al., 1978). Its distribution is strongly associated with the availability of flowering plants and appropriate nesting sites. This subspecies has been used in the Arabian Peninsula for beekeeping since 2,000 BA (Alqarni et al., 2011). While still poorly studied, *jemenitica* is described as specifically adapted to the ecological conditions of Yemen and surrounding regions (Al-Ghamdi and Nuru, 2013b). Phenotypically, *jemenitica* is among the smallest honey bee subspecies, typically displaying black or dark brown coloration, which may represent an adaptation for conserving energy and resources in arid environments (Ruttner, 1988). Other studies reported *jemenitica* as almost the size of *Apis cerana* (Oldroyd and Wongsiri, 2006) with a yellow abdomen and grey to brown bands (Al-Ghamdi and Nuru, 2013b). This suggests potential wide phenotypical and genetic diversity within *jemenitica* populations. Behaviorally, *jemenitica* is known for its relatively calm nature, making it easier to manage during beekeeping activities (Al-Ghamdi and Nuru, 2013b). Like African subspecies, *jemenitica* exhibits a tendency to swarm, which is a natural reproductive strategy. *Jemenitica* bees are efficient foragers, capable of sourcing nectar and pollen from various plants, including those that thrive in arid conditions. Their foraging behavior is adapted to maximize resource utilization in environments with limited floral availability (Al-Ghamdi and Nuru, 2013a). This subspecies has developed several physiological adaptations to cope with high temperatures, such as more efficient thermoregulation and behaviors that minimize exposure to heat. The foraging quality and efficiency of *jemenitica* in its native range of distribution make it a vital pollinator for many native plants, contributing to local biodiversity and the health of ecosystems.

Beekeeping practices in the regions of *jemenitica* often incorporate traditional methods passed down through generations. It is estimated, at least for the *jemenitica* populations of Saudi Arabia, that 70% or more are kept in traditional hives, often made of palm tree trunks, clay pots, and simple mud (Al-Ghamdi and Nuru, 2013b). Like many honey bee species, *jemenitica* faces threats from habitat loss, pesticide use, climate change, diseases, and the importation of foreign subspecies to its native repartition areas. Protecting natural habitats, promoting sustainable agricultural practices, and raising awareness of the importance of this subspecies are crucial for its conservation.

Honey bee diversity worldwide was first evaluated using morphological traits, including tergite pigmentation, body and wing dimensions, pilosity, tomentum width, cubital index, and proboscis length (Ruttner, 1988; Ftayeh et al., 1994; Meixner et al., 2007; Ruttner et al., 1978; Kauhausen-Keller et al., 1997; Kandemir et al., 2000; Arias et al., 2006; Alburaki and Alburaki, 2008). Four evolutionary lineages of honey bees were identified based on morphological traits: the West Mediterranean lineage (M), North Mediterranean lineage (C), African lineage (A), and Oriental lineage (O) (Ruttner, 1988; Ruttner et al., 1978; Rinderer, 1986). Although some recent studies have reported discrepancies, all four morphometrical lineages have been molecularly confirmed through mitochondrial and microsatellite markers (Smith and Brown, 1988; Smith and Brown, 1990; Cornuet et al., 1991; Garnery et al., 1992; Garnery et al., 1993; Estoup et al., 1995; Moritz et al., 1994; Garnery et al., 1998a; Garnery et al., 1998b; Meixner et al., 2000; Smith et al., 1991; Bouga et al., 2005). There are still unresolved discrepancies between mitochondrial DNA (mtDNA) and nuclear DNA analyses, particularly concerning the O and Y lineages (Ilyasov et al., 2020). For instance, the Y lineage has been reported to extend into the southern Arabian Peninsula and the Horn of Africa (Dogantzis and Zayed, 2019). However, this lineage has not been reported using morphometric traits or mtDNA markers (Ruttner, 1988; Ruttner et al., 1978; Kandemir et al., 2006). Currently, 33 honey bee subspecies, including *jemenitica*, have been identified in their native regions across Europe, Western Asia, and Africa (Sheppard and Meixner, 2003; Ruttner, 1988; Chen et al., 2016; Ilyasov et al., 2020).

The mitochondrial DNA (mtDNA) is a valuable tool in phylogeography and genetic studies due to its low or no recombination, relatively high evolutionary rate, conserved structure, and uniparental heredity (Avise et al., 1987; Harrison, 1989; Avise, 2000). The cytochrome *b* (Cyt *b*) gene is an essential component of the mitochondrial genome in bees and other organisms (Toparslan et al., 2020; Tobe et al., 2010). This gene encodes a protein critical in the electron transport chain, vital for cellular respiration and energy production in the mitochondria. The cyt *b* gene is often studied for its genetic variability among honey bee lineages and subspecies (Crozier and Crozier, 1992; Alburaki et al., 2011; Pinto et al., 2004). Its variation can help us understand honey bee evolutionary relationships, population structure, and local adaptation to specific environments. Variations in the cyt *b* gene can also offer insights into the genetic health of honey bee populations by assessing the impact of environmental stressors and diseases on bee populations. Coupled with other markers, the cyt *b* gene can serve as a valuable tool in bee research, aiding in understanding the genetics, evolution, and conservation of honey bees. Similar to the cytochrome *b* (cyt *b*), cytochrome *c* oxidase I (COI) is a mitochondrial coding gene commonly used to investigate the genetic diversity of honey bee subspecies (Kandemir et al., 2006).

The DraI mtDNA COI-COII test, referred to as (DmCC), is a widely used mitochondrial test in honey bee genetics (Garnery et al., 1993; Smith and Brown, 1990; Cornuet et al., 1991). This test is conducted on the mtDNA intergenic region situated between both cytochrome *c* oxidase I and II. This region’s polymorphisms can determine, to a great extent, the evolutionary lineage, subspecies, and haplotype, rendering this test one of the most comprehensive and efficient tests to explore honey bee genetic diversity (Alburaki et al., 2011; Rortais et al., 2011; Arias and Sheppard, 1996; De la Rua et al., 1998; Franck et al., 2001; Palmer et al., 2000; Loucif-Ayad et al., 2014; Franck et al., 2000; Hall and Smith, 1991; Abrahamovich et al., 2007; El-Niweiri and Moritz, 2008).

The genetic diversity of *jemenitica* has marginally been explored during the last decades (Alghamdi et al., 2012; Alattal et al., 2014b). This study aims to evaluate the genetic diversity of *jemenitica* using mitochondrial DNA markers and to assess its evolutionary relationships with neighboring subspecies. We hypothesize that *jemenitica* populations exhibit significant genetic diversity due to their broad geographic range and unique adaptations to various arid environments. Our data identified novel haplotypes, indicated structural differences between *jemenitica* and other neighboring subspecies, and a wider *jemenitica* genetic diversity within the African lineage than previously presumed.

## 2 Materials and methods

### 2.1 Honey bee samples

**FIGURE 1:**
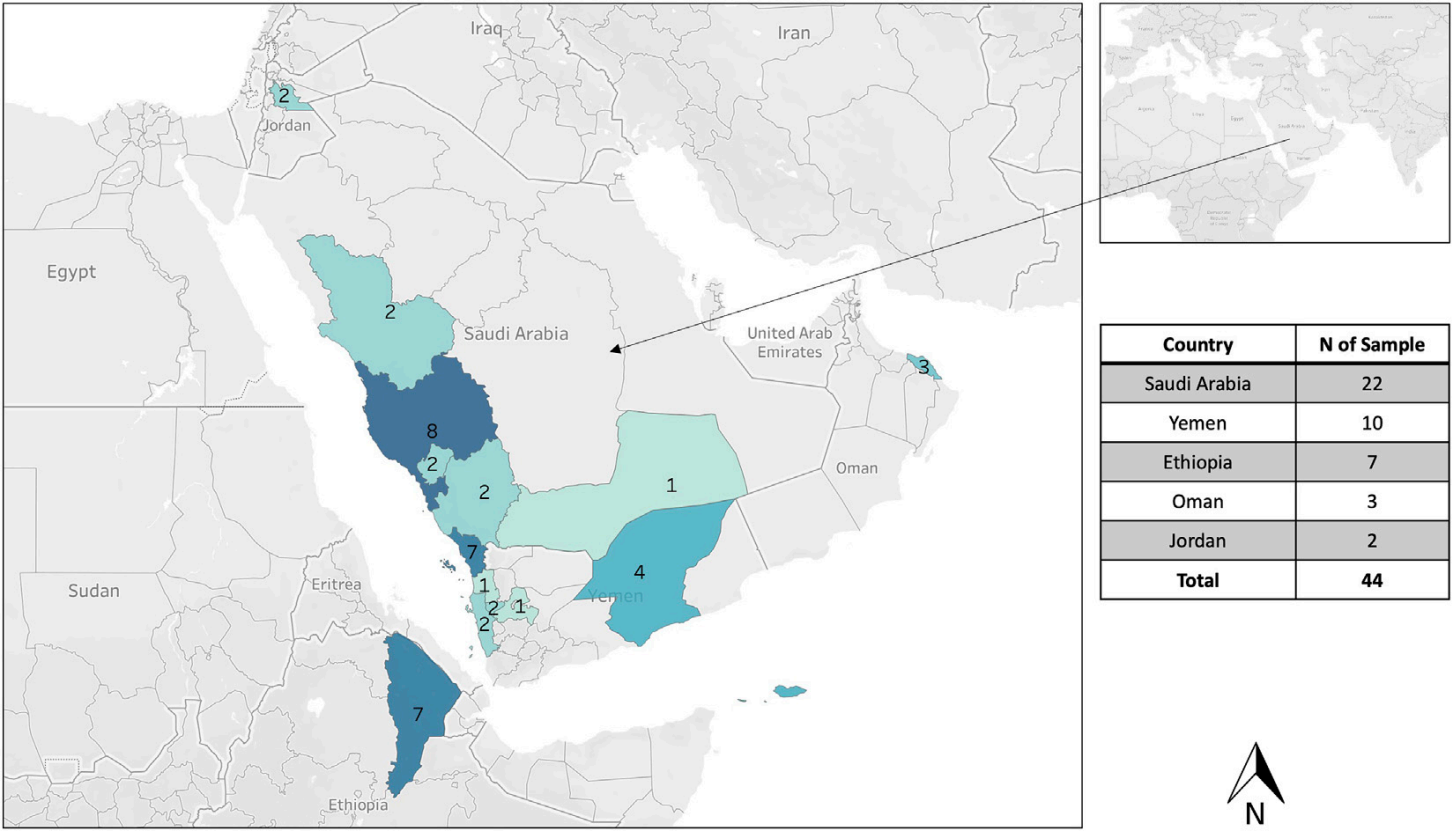
Geographical distribution of the sampling from five different countries: Saudi Arabia, Yemen, Sultanate of Oman, Ethiopia, and Jordan. One honey bee sample was collected per hive, and 44 samples were analyzed for the cyt *b*, 15 samples for the COI gene, and the intergenic COI-COII region.

A total of 44 honey bee samples were sampled from five different countries: 1- Saudi Arabia (n = 22), Yemen (10), Ethiopia (7), Oman (3), and Jordan (2), Figure 1; Table 1. The sample size of 44 individuals from five countries was limited by logistical constraints but deemed sufficient to capture a broad representation of *jemenitica* populations across the study regions. One honey bee worker was sampled per colony. The region and city of each sample are detailed in Table 1. All samples originated from small-scale traditional apiaries, mainly belonging to stationary beekeepers. All samples (n = 44) were analyzed for cyt *b*, and 15 samples were studied for the COI gene and the intergenic region COI-COII. Worker bee samples were sampled in absolute ethanol and stored at 20°C at the Bee Research Unit at King Saud University for further downstream analyses. Due to mid-experiment technical difficulties and a change of institutions by the principal investigator, only 15 samples of the original 44 samples were available to run for COI and COI-COII regions.

**TABLE 1:** Geographic distribution and details of *A. mellifera jemenitica* samples analyzed for the cyt *b*.

Code	Country	Region/City	N	Cyt *b* bp	NCBI	SG	Blast	%	Subspecies
Eth1	Ethiopia	Afar/Eastern part	1	359	PQ460982	1	MN714161	100	*Apis m. jemenitica*
Eth2	Ethiopia	Afar/Eastern part	1	359	PQ460983	1	MN714161	100	*Apis m. jemenitica*
Eth3	Ethiopia	Afar/Eastern part	1	359	PQ460984	1	MN714161	100	*Apis m. jemenitica*
Eth4	Ethiopia	Afar/Eastern part	1	359	PQ460985	4	MT572355	100	*Apis m. simensis*
Eth5	Ethiopia	Afar/Eastern part	1	359	PQ460986	4	MT572355	100	*Apis m. simensis*
Eth6	Ethiopia	Afar/Eastern part	1	359	PQ460987	4	MT572355	100	*Apis m. simensis*
Eth7	Ethiopia	Afar/Eastern part	1	359	PQ460988	8	KY464958	99.7	*Apis m. lamarckii*
Jor1	Jordan	Amman	1	359	PQ460989	6	KP163643	99.7	*Apis m. syriaca*
Jor2	Jordan	Amman	1	359	PQ460990	6	KP163643	99.7	*Apis m. syriaca*
Oma1	Oman	Al-Rustaq/Amak	1	359	PQ460991	1	MN714161	100	*Apis m. jemenitica*
Oma2	Oman	Al-Rustaq/Al-Jafr	1	359	PQ460992	1	MN714161	100	*Apis m. jemenitica*
Oma3	Oman	Al-Rustaq/Al-Ghashb	1	359	PQ460993	1	MN714161	100	*Apis m. jemenitica*
Sau1	Saudi Arabia	Najran	1	359	PQ460994	3	KR010390	100	*Apis m. jemenitica*
Sau2	Saudi Arabia	Aseer	1	359	PQ460995	1	MN714161	100	*Apis m. jemenitica*
Sau3	Saudi Arabia	Aseer	1	359	PQ460996	1	MN714161	100	*Apis m. jemenitica*
Sau4	Saudi Arabia	Jazan	1	359	PQ460997	5	KM433625	99.7	*Apis m. Iberiensis*
Sau5	Saudi Arabia	Jazan	1	359	PQ460998	2	KY464958	100	*Apis m. lamarckii*
Sau6	Saudi Arabia	Jazan	1	359	PQ460999	2	KY464958	100	*Apis m. lamarckii*
Sau7	Saudi Arabia	Jazan	1	359	PQ461000	2	KY464958	100	*Apis m. lamarckii*
Sau8	Saudi Arabia	Jazan	1	359	PQ461001	1	MN714161	100	*Apis m. jemenitica*
Sau9	Saudi Arabia	Jazan	1	359	PQ461002	2	KY464958	100	*Apis m. lamarckii*
Sau10	Saudi Arabia	Jazan	1	359	PQ461003	2	KY464958	100	*Apis m. lamarckii*
Sau11	Saudi Arabia	Al Baha	1	359	PQ461004	1	MN714161	100	*Apis m. jemenitica*
Sau12	Saudi Arabia	Al Baha	1	359	PQ461005	1	MN714161	100	*Apis m. jemenitica*
Sau13	Saudi Arabia	Taif	1	359	PQ461006	1	MN714161	100	*Apis m. jemenitica*
Sau14	Saudi Arabia	Taif	1	359	PQ461007	1	MN714161	100	*Apis m. jemenitica*
Sau15	Saudi Arabia	Taif	1	359	PQ461008	1	MN714161	100	*Apis m. jemenitica*
Sau16	Saudi Arabia	Taif	1	359	PQ461009	1	MN714161	100	*Apis m. jemenitica*
Sau17	Saudi Arabia	Taif	1	359	PQ461010	1	MN714161	100	*Apis m. jemenitica*
Sau18	Saudi Arabia	Al Madinah	1	359	PQ461011	2	KY464958	100	*Apis m. lamarckii*
Sau19	Saudi Arabia	Al Madinah	1	359	PQ461012	2	KY464958	100	*Apis m. lamarckii*
Sau20	Saudi Arabia	Taif	1	359	PQ461013	1	MN714161	100	*Apis m. jemenitica*
Sau21	Saudi Arabia	Taif	1	359	PQ461014	2	KY464958	100	*Apis m. lamarckii*
Sau22	Saudi Arabia	Taif	1	359	PQ461015	2	KY464958	100	*Apis m. lamarckii*
Yem1	Yemen	Hajjah	1	359	PQ461016	1	MN714161	100	*Apis m. jemenitica*
Yem2	Yemen	Al-Hodeidah	1	359	PQ461017	1	MN714161	100	*Apis m. jemenitica*
Yem3	Yemen	Al-Hodeidah	1	359	PQ461018	1	MN714161	100	*Apis m. jemenitica*
Yem4	Yemen	Al-Mhweit	1	359	PQ461019	11	MT572355	99.7	*Apis m. simensis*
Yem5	Yemen	Al-Mhweit	1	359	PQ461020	7	KR010390	99.7	*Apis m. jemenitica*
Yem6	Yemen	Hadramout/Seyon	1	359	PQ461021	2	KY464958	100	*Apis m. lamarckii*
Yem7	Yemen	Hadramout/Seyon	1	359	PQ461022	10	MN714161	99.7	*Apis m. jemenitica*
Yem8	Yemen	Sana’a	1	359	PQ461023	9	MN119925	99.7	*Apis m. unicolor*
Yem9	Yemen	Hadramout/Seyon	1	359	PQ461024	2	KY464958	99.7	*Apis m. lamarckii*
Yem10	Yemen	Socotra Archipelago	1	359	PQ461025	1	MN714161	100	*Apis m. jemenitica*

Number of samples (N), and sequence groups (SG). (NCBI) is the accession number assigned to each sequence, (Blast) is the accession number of the highest matching score (%) with a reference sequence and the subspecies assigned to the reference sequence.

### 2.2 DNA extraction

Genomic DNA was extracted from the thoraxes of honey bee workers using the DNeasy^®^ Blood and Tissue Kit (Qiagen, Germany), following the manufacturer’s protocol. Briefly, thoraxes were dissected using a scalpel and forceps and kept in 2 mL microcentrifuge tubes. Two glass beads and 180 μL ATL buffer were added to the tubes, and tissues were homogenized for 5 min at speed 10 (Equivalent to 6,000 cycles/minute) in a Bullet Blender (Next Advance, Inc., Averill Park, NY). A 20 μL of Proteinase K (10 mg/mL) was added per sample, vortexed, and incubated at 56°C overnight. Tube contents were transferred to DNeasy mini spin column for a DNA column extraction according to the manufacturer’s protocol. DNA extracts were stored at −20°C for further use.

### 2.3 Amplification of the coding genes cyt *b* and COI

All samples (n = 44) were amplified at both ends for the cyt *b* gene using the universal honey bee set of primers: Cytb-F: 5′- TAT​GTA​CTA​CCA​TGA​GGA​CAA​ATA​TC-3′ and Cytb-R: 5′-ATT​ACA​CCT​CCT​AAT​TTA​TTA​GGA​AT-3′ (Crozier et al., 1991; de Oliveira Francisco et al., 2001; Sheppard et al., 1994; Alburaki et al., 2011). Fifteen samples among the 44 were amplified for the COI gene using the following set of primers: COI-F: 5′- TCT​ATA​CCA​CGA​CGT​TAT​TC-3′ and COI-R: 5′- GAT​CAA​TAT​CAT​TGA​TGA​CC -3′ (Smith et al., 1997). The PCR reaction was conducted in a 12 µL reaction composed of 6 µL BioRad Master Mix, 0.5 µL of each primer (10 µM), and 5 µL of nuclease-free water. The PCR cycling for both genes was 95°C for 3 min, followed by 35 cycles of (95°C for 10 s, 55°C for 30 s) and a permanent hold at 4°C. The standard two-step PCR amplification was efficient and produced optimal bands, eliminating the need for a final extension phase. Negative controls were included in all PCR reactions to ensure the absence of contamination, and positive controls were used to verify amplification success. PCR products were verified by electrophoresis on a 1.5% agarose gel.

### 2.4 Amplification of the intergenic non-coding region COI-COII

The intergenic non-coding region of the mtDNA located between the cytochrome *c* oxidase I and II was studied on the same fifteen samples evaluated for the COI gene. This region was amplified at both ends using the traditional set of primers: E2-F: 5′-GGC​AGA​ATA​AGT​GCA​TTG-3′, H2-R: 5′-CAA​TAT​CAT​TGA​TGA​CC-3′ (Alburaki et al., 2011; Rortais et al., 2011; Achou et al., 2015; Meixner et al., 2000; Garnery et al., 1993; Franck et al., 2001). The PCR reaction for this set of primers was carried out in a 26 µL reaction composed of 12.5 µL of Promega’s 1X Taq buffer, 1.9 mM MgCl2, 0.2 mM of each dNTP, 0.14 µM of each primer, 16.5 µL of nuclease-free water, 1.25 U/reaction of Taq, and 1.4 µL of DNA. PCR cycling consisted of the following parameters: 95°C for 2 min, followed by 30 cycles of (95°C for 30 s, 54°C for 30 s, 72°C for 30 s), and 72°C extension phase for 10 min. Negative controls were included in all PCR reactions to ensure the absence of contamination. Similar to both previous genes, amplicons were run on 1.5% agarose gel for size and quality checks.

### 2.5 Amplicon sequencing and alignment

PCR products were purified using Applied Biosystems ExoSAP-IT kit and sequenced at both ends using an automated sequencer (Applied Biosystems 3730XL DNA Analyzer, United States). Sequencing quality was assessed using (3730XL Data Collection Software 5), with ambiguous bases trimmed and only high-quality reads included in the analysis. Each sample’s forward and reverse sequences for all three regions (Cyt *b*, COI, and COI-COII) were paired after reversing the reverse sequence, aligned, and manually checked using Geneious Prime^®^ 2024.0.7 (Drummond et al., 2011). Nucleotide ambiguities at both sequence ends were trimmed, and all sequences generated from this study (n = 74) were deposited at the National Center for Biotechnology Information (NCBI) GenBank.

### 2.6 Data analysis

Geographical mappings of the populations’ distributions were conducted using the Tableau Public platform (https://www.tableau.com). Sequence alignment, verification, generation of FASTA files, and phylogenetic analysis were carried out using Geneious Prime^®^ 2024.0.7 (https://www.geneious.com). Sequences of both coding genes (Cyt *b* and COI) were separately aligned by MUSCLE standard alignment algorithm (Edgar, 2004), and reconfirmed by Geneious alignment using a Cost Matrix of 65% similarity (5.0/-4.0) with a “Global alignment with free end gaps” option, then trimmed at both ends to eliminate non-identified nucleotides. However, the COI-COII non-coding sequences (n = 15) were first sorted out according to the genetic structure and length of the intergenic region (Q, P, P0, PQ_x_, P0Q_x_), in which (x) is a tandem repeat of the Q fragment, and analyzed following the steps described in previous studies (Madella et al., 2020; Alburaki et al., 2023). All sequences were blasted against the NCBI databases, and the highest blasting scores were reported in this study, along with their reference sequences. Novel COI-COII haplotypes were named according to the universal nomenclature detailed in a recent study (Alburaki et al., 2023).

Generation of haplotype networks and computation of nucleotide diversity and Tajima’s statistic were conducted in the R environment (R Core Team, 2022) (v. 2024.09.0) using molecular-variance parsimony available in the libraries: “ape” and “pegas” (Paradis, 2010; Paradis and Schliep, 2019). Maximum-likelihood phylogenetic trees were conducted using the Tamura-Nei distance model (Tamura and Nei, 1993) employing the “PHYML” plugin (Guindon et al., 2010) with default parameters: Number of bootstraps = 100, automatic estimation of transition ratio, proportion of invariable sites, and Gamma distribution parameter, with a default number of substitution rate categories of four. This study prioritized the maximum likelihood method for constructing phylogenetic trees, given the limited number of sequences available and the assumption of varying evolutionary rates among the samples (Zou et al., 2024). When relevant, genetic trees were rooted using *A. cerana* sequences (FJ229480) or genetic clades from distant non-African lineages. Only NCBI-verified and accurate reference sequences of other subspecies were used in this study, including previously published *jemenitica* haplotypes from the same studied regions (Supplementary Table S1). For some genes, the absence of neighboring reference sequences (e.g., *simensis*, *litorea*, *monticola*) has restricted the scope of our analyses.

## 3 Results

### 3.1 Phylogenetic analysis of cyt *b* gene

**FIGURE 2:**
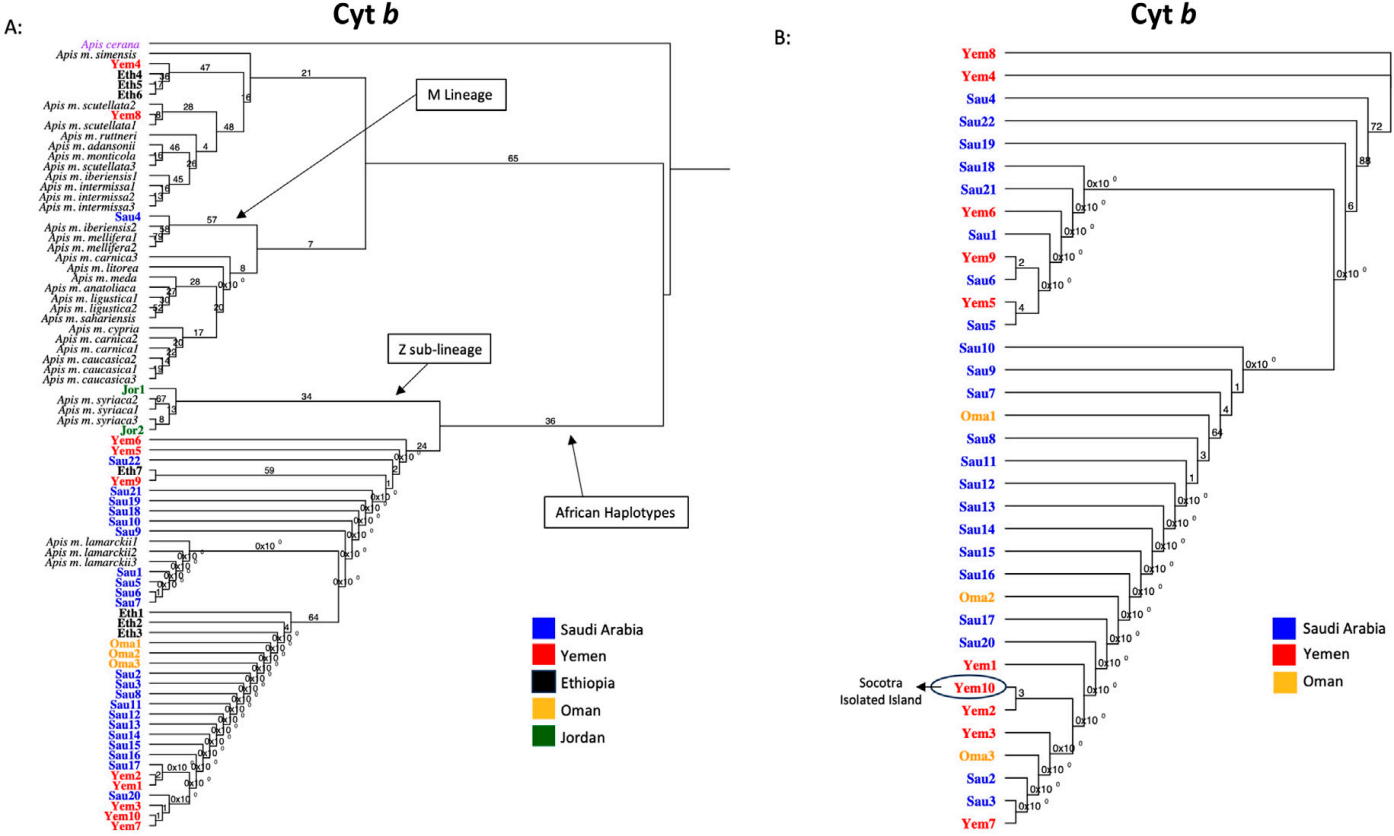
Maximum-likelihood phylogenetic tree of *A. mellifera jemenitica* samples based on cyt *b* sequences. Bootstrap values are shown on major nodes. Studied samples are displayed in bold font and color-coded; the rest are NCBI reference samples of major honey bee subspecies. Trees are rooted with *Apis cerana* as an outgroup species. **(A)** Represents an inter-lineage analysis. **(B)** Intra-sample analysis.

All forty-four studied samples from five countries (Figure 1) were amplified for the cyt *b* gene with a 359 bp length after trimming ambiguous nucleotides at both ends, Table 1. Out of 44 sequences, 35 blasted at 100% with reference samples representing three different subspecies: *jemenitica*, *simensis*, and *lamarckii*. The nine other samples blasted at 99.7% as follows: 1- Jordan (n = 2): *syriaca*, 2- Ethiopia (n = 1): *lamarckii*, 3- Saudi Arabia (n = 1): *iberiensis*, and 4- Yemen (n = 5): *jemenitica*, *simensis*, *unicolor*, *lamarckii*, Table 1. A total of 11 cyt *b* sequence groups were recorded, characterizing six different subspecies, Table 1. In the phylogenetic analysis, most samples clustered in an independent African lineage (A) clade with *lamarckii* and separated from the African sub-lineage (Z) with a 36-bootstrap value, Figure 2A. Samples from Jordan, the native region of *syriaca*, clustered with *syriaca* references (Figure 2A). Two Yemeni samples (Yem4, 8) and three Ethiopian samples (Eht4, 5, 6) showed closer genetic proximity to *simensis* and *scutellata*, Figure 2A. The intra-sample phylogenetic tree, rooted with *A. cerana* separated (33-bootstrap) the Jordanian samples (Jor1, 2) from the rest as well as Omani samples and one single Ethiopian sample (Eth1) with a 64-bootstrap value, Figure 2B. The sample (Sau4), which clustered within the M lineage (Figure 2A), remained quite distant (90-bootstrap) from other samples, Figure 2B. All forty-four cyt *b* sequences are available at NCBI GenBank under accession numbers PQ460982 to PQ461025, Table 1.

### 3.2 COI sequence analysis

**FIGURE 3:**
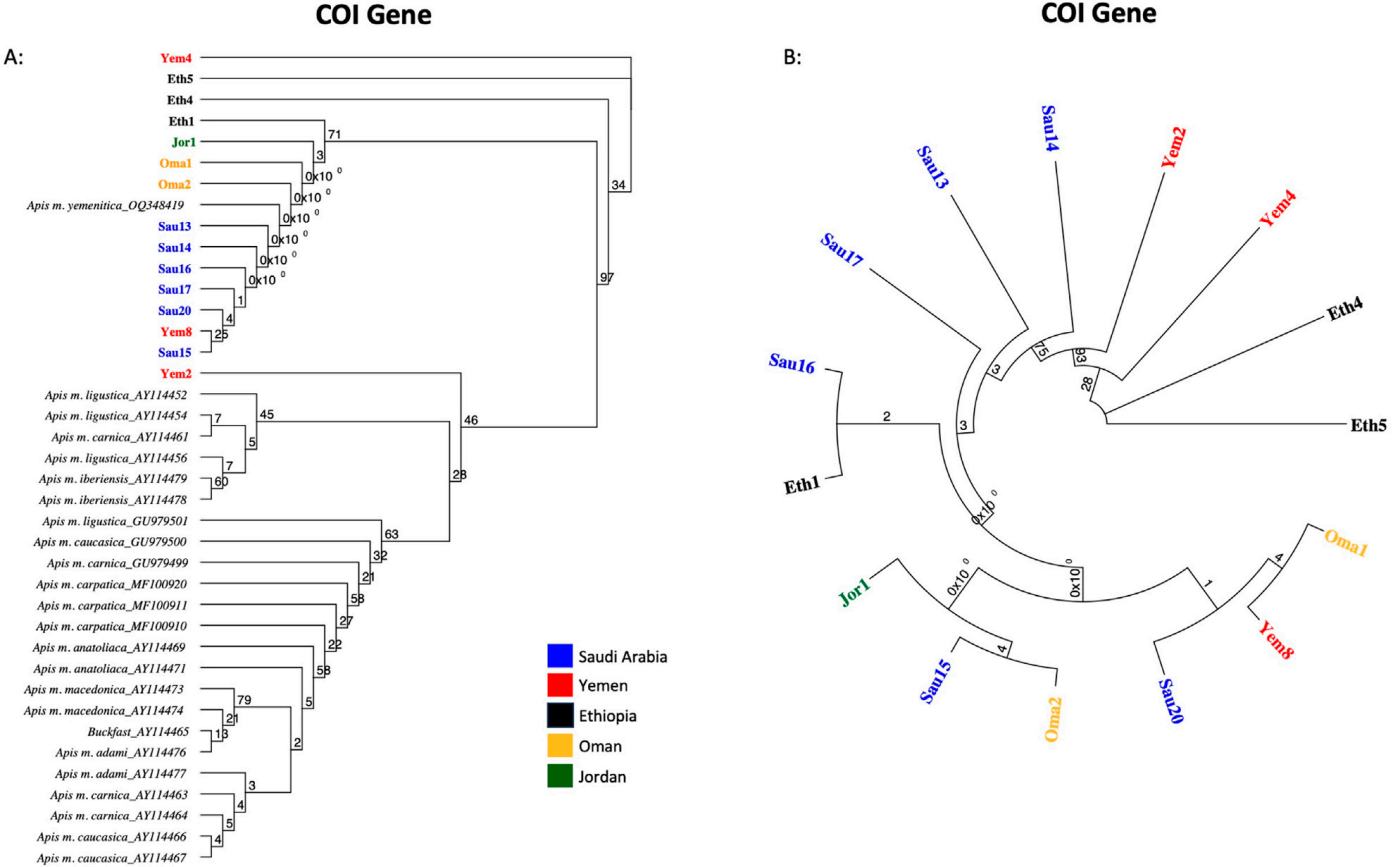
Maximum-likelihood phylogenetic tree of *A. mellifera jemenitica* samples based on COI sequences. Bootstrap values are shown on major nodes. Samples of this study are displayed in bold font and color-coded; the rest are NCBI reference samples of major honey bee subspecies. **(A)** Represents an inter-lineage analysis, **(B)** intra-sample analysis.

Fifteen samples were sequenced for the COI coding gene, Table 2. The verified and retained sequence length of the COI gene was 218 bp. NCBI-verified reference sequences in African subspecies for the COI gene were much less abundant, which restricted the scope of this analysis. Five sequence groups were recorded within the studied samples, Table 2. All sequences except one (Sau15) blasted at 100% with reference sequences of various subspecies: *jemenitica*, *syriaca*, *lamarckii*, *simensis*, *scutellata*, *monticola*, *capensis*, *mellifera* and *ligustica*, Table 2. Sau15 exhibited a SNP (C replacing T) at the gene end (155 bp). Based on the sequences of this gene, Yem4 and Eth5 are the most genetically distant among the studied samples, Figure 3A. Eth4, however, formed an independent cluster with a bootstrap value of 34. In contrast, other samples formed a solid clade (97-bootstrap) with a *jemenitica* reference (OQ348419) away from other subspecies belonging to the C, O, and M lineages, Figure 3A. In the intra-sample phylogenetic analysis, Eth4 and Eth5 were the most distant samples, followed by Yem4 (28-bootstrap), Yem2 (93-bootstrap), and Sau14 (75-bootstrap), while the rest of the samples exhibited high genetic similarity with weak bootstrap values, Figure 3B. All fifteen COI sequences are available at NCBI GenBank under accession numbers PQ461026 to PQ461040, Table 2.

**TABLE 2:** Geographic distribution and details of *A. mellifera jemenitica* samples analyzed for the COI gene.

Code	Country	Region/City	N	COI gene bp	NCBI	SG	Blast	%	Subspecies
Eth1	Ethiopia	Afar/Eastern part	1	218	PQ461026	1	NC051935	100	*jemenitica, syriaca, lamarckii, mellifera*
Eth4	Ethiopia	Afar/Eastern part	1	218	PQ461027	2	MN585108	100	*simensis, mellifera*
Eth5	Ethiopia	Afar/Eastern part	1	218	PQ461028	2	MN585108	100	*simensis, mellifera*
Jor1	Jordan	Amman	1	218	PQ461029	1	MN714161	100	*jemenitica, syriaca, lamarckii, mellifera, ligustica*
Oma1	Oman	Al-Rustaq/Amak	1	218	PQ461030	1	MN714161	100	*jemenitica, syriaca, lamarckii, mellifera, ligustica*
Oma2	Oman	Al-Rustaq/Al-Jafr	1	218	PQ461031	1	MN714161	100	*jemenitica, syriaca, lamarckii, mellifera, ligustica*
Sau13	Saudi Arabia	Taif	1	218	PQ461032	1	MN714161	100	*jemenitica, syriaca, lamarckii, mellifera, ligustica*
Sau14	Saudi Arabia	Taif	1	218	PQ461033	1	MN714161	100	*jemenitica, syriaca, lamarckii, mellifera, ligustica*
Sau15	Saudi Arabia	Taif	1	218	PQ461034	3	MN714161	99.5	*jemenitica, syriaca, lamarckii, mellifera, ligustica*
Sau16	Saudi Arabia	Taif	1	218	PQ461035	1	MN714161	100	*jemenitica, syriaca, lamarckii, mellifera, ligustica*
Sau17	Saudi Arabia	Taif	1	218	PQ461036	1	MN714161	100	*jemenitica, syriaca, lamarckii, mellifera, ligustica*
Sau20	Saudi Arabia	Taif	1	218	PQ461037	1	MN714161	100	*jemenitica, syriaca, lamarckii, mellifera, ligustica*
Yem2	Yemen	Al-Hodeidah	1	218	PQ461038	4	KJ601784	100	*scutellata, monticola, capensis, mellifera*
Yem4	Yemen	Al-Mhweit	1	218	PQ461039	1	MN585108	100	*simensis, mellifera*
Yem8	Yemen	Sana’a	1	218	PQ461040	5	KP163643	100	*syriaca, jemenitica*

Sample size (N) and sequence groups (SG). (NCBI) is the accession number assigned to each sample, and (Blast) is the accession number of the highest matching reference sequence and its blasting percentage with the subspecies assigned to the reference sequence. Additional subspecies reference sequences with 100% blast were also reported.

### 3.3 COI-COII structure, haplotype and phylogeny

**FIGURE 4:**
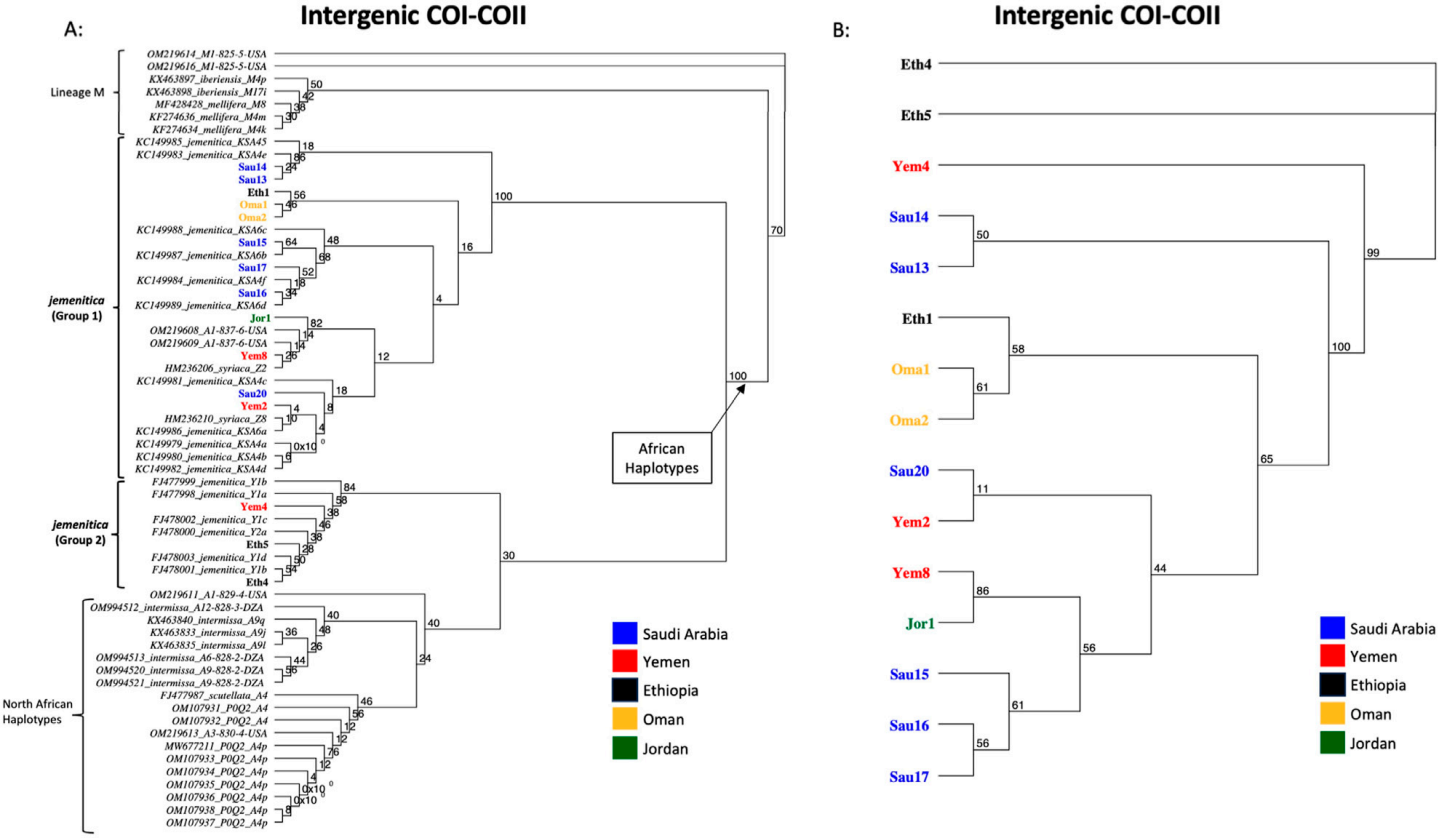
Maximum-likelihood phylogenetic tree of *A. mellifera jemenitica* samples based on COI-COII sequences. Bootstrap values are shown on major nodes. Samples of this study are displayed in bold font and color-coded; the rest are NCBI reference sequences from both African (A) and West Mediterranean lineages (M) with a P0Q2 structure. **(A)** Represents an inter-lineage analysis, **(B)** intra-sample analysis. Numbers on branches represent the bootstrap support values.

The same fifteen samples studied for COI were sequenced for their intergenic COI-COII regions. The COI-COII sequences were captured, varying in length from 818 to 839 bp, Table 3. All sequences exhibited one of the typical African COI-COII structures (P0Q2). Interestingly, a deletion of 18 pb was identified in the P0 element of three samples (Eth4, Eth5, Yem4), Table 3. This distinct and significant deletion matches those found in reference *jemenitica* sequences, all collected from Ethiopia (FJ477998, FJ477999, FJ478000, FJ478001, FJ478002, FJ478003), Table 3. A total of 13 COI-COII haplotypes were identified, among which eight were novel, Table 3. Six samples fully blasted (100%) with previously identified *jemenitica* haplotypes from Saudi Arabia (KSA4e, KSA6b, KSA6d, KSA4f) and one African haplotype from the United States (A1-818-6-USA), Table 3. The highest blasting scores (99.4%–99.9%) for the novel haplotypes were all with *jemenitica* references and one sample (Yem8) with *syriaca*, Table 3. Only reference sequences of the same COI-COII structure (P0Q2) were selected and run in the phylogenetic analysis. The studied samples clustered into two distinct clades supported by a 100-bootstrap value, Figure 4A. Group 1 comprised most samples clustering with reference sequences of *jemenitica*, *syriaca*, and Group 2, representing previously recorded Ethiopian haplotypes. The latter branch was separated from other North African subspecies and supported with a 30-bootstrap value, Figure 4A. The intra-sample COI-COII variation is higher than what was seen for the two other genes. Eth4 and Eth5 were the most distant haplotypes, followed by Yem4 (99-bootstrap) and Sau14 and Sau13 (Same haplotype; 100-bootstrap), Figure 4B. The novel Omani haplotype (A1-838-6-OMN) showed greater similarity to Eth1, another novel haplotype (A1-839-6-ETH) identified in this study (Figure 4B). All fifteen COI-COII sequences are available at NCBI GenBank under accession numbers PQ461041 to PQ461055, Table 3.

**TABLE 3:** Geographic distribution and details of *A. mellifera jemenitica* samples analyzed for the COI-COII intergenic region.

Code	Country	Region/City	N	bp	COI-COII structure	L	Haplotype	HG	NCBI	Blast/Haplotype	%	Subspecies
Eth1*	Ethiopia	Afar/Eastern part	1	839	P0Q2	A	A1-839-6-ETH	11	PQ461041	KC149979/KSA4a	99.6	*Apis m. jemenitica*
Eth4*	Ethiopia	Afar/Eastern part	1	820	P0Q2 (Deletion 18 bp in P0)	A	A2-820-5-ETH	3	PQ461042	FJ478003/Y2d	99.6	*Apis m. jemenitica*
Eth5*	Ethiopia	Afar/Eastern part	1	818	P0Q2 (Deletion 18 bp in P0)	A	A3-818-5-ETH	4	PQ461043	FJ78000/Y2a	99.9	*Apis m. jemenitica*
Jor1	Jordan	Amman	1	837	P0Q2	A	A1-837-6-USA	1	PQ461044	OM219608	100	African
Oma1*	Oman	Al-Rustaq/Amak	1	838	P0Q2	A	A1-838-6-OMN	13	PQ461045	KC149983/KSA4e	99.7	*Apis m. jemenitica*
Oma2*	Oman	Al-Rustaq/Al-Jafr	1	838	P0Q2	A	A1-838-6-OMN	13	PQ461046	KC149983/KSA4e	99.7	*Apis m. jemenitica*
Sau13	Saudi Arabia	Taif	1	838	P0Q2	A	KSA4e	5	PQ461047	KC149983	100	*Apis m. jemenitica*
Sau14	Saudi Arabia	Taif	1	838	P0Q2	A	KSA4e	5	PQ461048	KC149983	100	*Apis m. jemenitica*
Sau15	Saudi Arabia	Taif	1	837	P0Q2	A	KSA6b	6	PQ461049	KC149987	100	*Apis m. jemenitica*
Sau16	Saudi Arabia	Taif	1	837	P0Q2	A	KSA6d	7	PQ461050	KC149989	100	*Apis m. jemenitica*
Sau17	Saudi Arabia	Taif	1	838	P0Q2	A	KSA4f	8	PQ461051	KC149984	100	*Apis m. jemenitica*
Sau20*	Saudi Arabia	Taif	1	838	P0Q2	A	A1-838-6-SAU	9	PQ461052	KC149979/KSA4a	99.9	*Apis m. jemenitica*
Yem2*	Yemen	Al-Hodeidah	1	836	P0Q2	A	A1-836-6-YEM	10	PQ461053	KC149979/KSA4a	99.7	*Apis m. jemenitica*
Yem4*	Yemen	Al-Mhweit	1	818	P0Q2 (Deletion 18 bp in P0)	A	A2-818-6-YEM	12	PQ461054	FJ478003/Y2b	99.4	*Apis m. jemenitica*
Yem8*	Yemen	Sana’a	1	836	P0Q2	A	A3-836-6-YEM	2	PQ461055	OM219608/Z2	98.8	*Apis m. syriaca*

(N) is the number of samples, (bp) amplified length of the COI-COII, (L) evolutionary lineage, and (HG) is the number of haplotype groups identified. (NCBI) are accession numbers assigned to the sequences of this study. Haplotypes were determined based on 100% blasting with NCBI reference sequences. Subspecies cited are those of the reference sequence (Blast/Haplotype) with the highest blasting scores. Novel haplotypes are marked with (*).

### 3.4 Haplotype diversity and network

**FIGURE 5:**
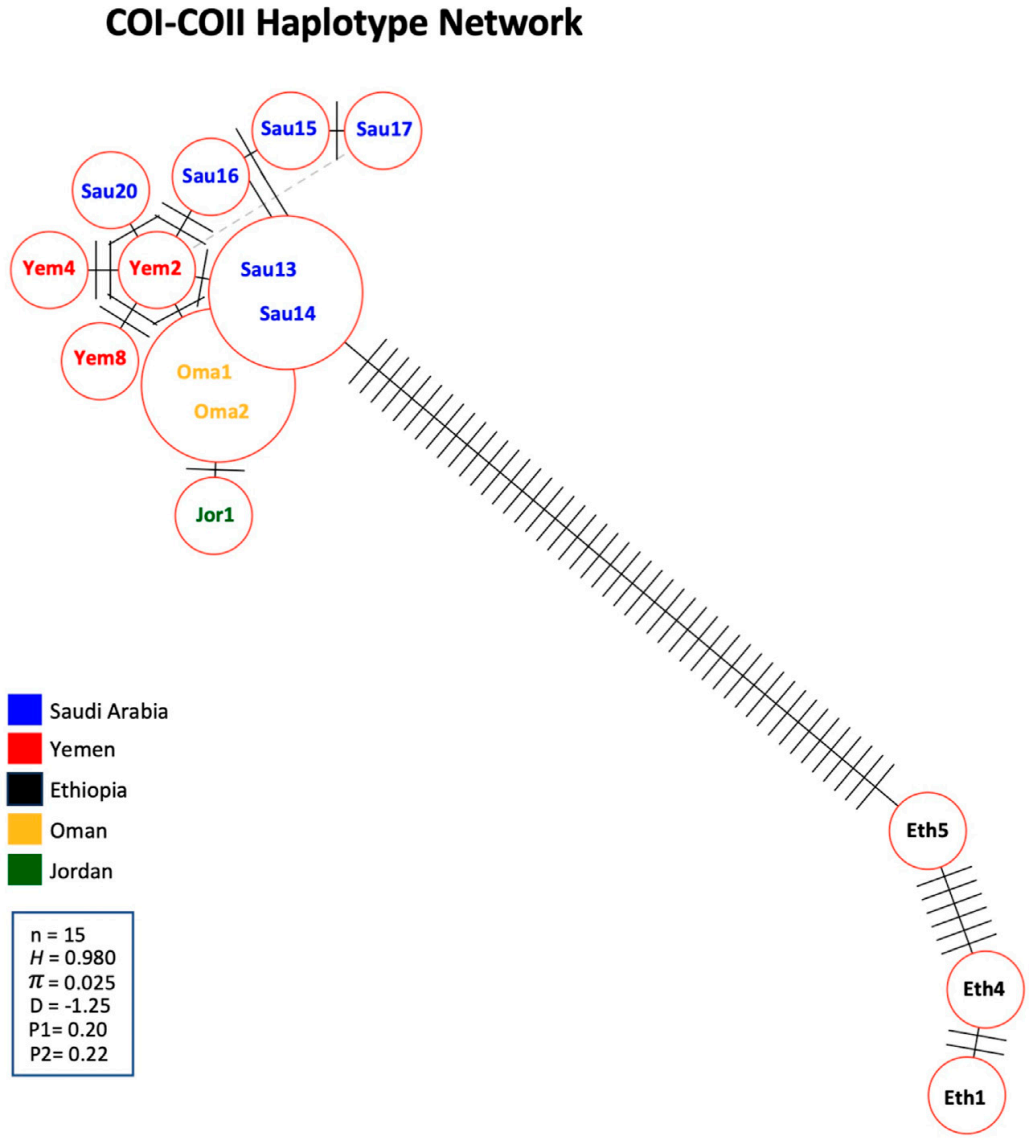
Haplotype network of the COI-COII intergenic region of the 15 studied samples. Number of samples (n), haplotype diversity (*H*), nucleotide diversity (π), Tajima’s statistic (D), Tajima’s *p*-values under normal (P1) and beta (P2) distributions. Perpendicular lines on branches represent nucleic changes. The dotted lines represent potential haplotype secondary networks.

A significant number of COI-COII haplotypes (n = 13) were identified in a relatively small population of 15 samples. The haplotype diversity (*H*) was 0.980, consistent with the corrected (*H)*, as no non-African COI-COII haplotypes were identified (Table 3; Figure 5). The nucleotide diversity (π) within the studied samples was (0.025) with a Tajima’s statistic (D = −1.25, *p* > 0.05) indicating no deviation from neutrality or significant selection activity, Figure 5. Interestingly, the COI-COII haplotype network suggests a significant genetic distance between Ethiopian haplotypes (Eth1, Eth4, Eth5) and the rest, Figure 5. All other haplotypes seem to have diverged from each other through (≥1) SNPs, Figure 5. Within this cluster, Yem2 occupied a central position from which other haplotypes may have diverged, Figure 5.

**FIGURE 6:**
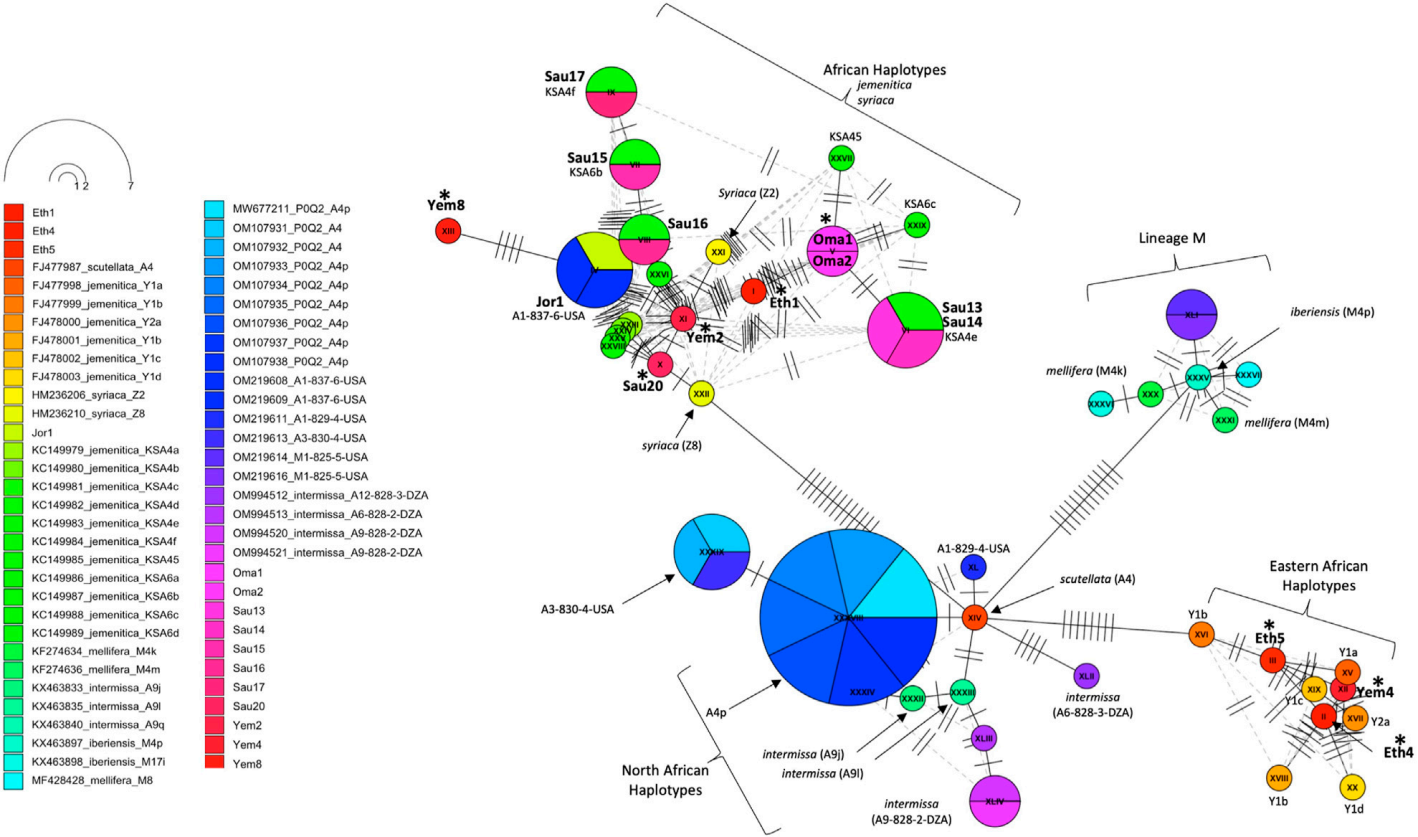
Haplotype network of the COI-COII intergenic region of 15 studied samples and 47 other reference sequences with P0Q2 structure. Studied samples are in bold font, with an asterisk for novel haplotypes. Subspecies and haplotypes are displayed for reference sequences when available. Haplotype references, which blasted 100% (Table 3), are shown below the studied samples. The dotted lines represent potential secondary haplotype networks. Perpendicular lines on branches represent nucleic changes.

An assorted number of (P0Q2) NCBI reference sequences (n = 47, Supplementary Table S1) from various subspecies were selected to study the inter-genetic diversity of our samples. The COI-COII haplotype network distinguished four groups of haplotypes: 1- Haplotypes of the M lineage (M4p, M4k, M4m) known to characterize both *mellifera* and *iberiensis* subspecies, 2- Eastern African haplotypes (Y1a, Y1b, Y1c, Y1d, Y2a) sampled from Ethiopia with which clustered three of the studied samples (Eth4, Eth5, Yem4), 3- North African haplotypes (A4p, A9i, A9l) characterizing *intermissa*, including two African haplotypes (A3-830-4-USA, A1-829-4-USA) recently identified in the United States, and 4- The majority of the Arabian Peninsula’s samples including those of Jordan clustering with reference samples of *syriaca* and previously identified *jemenitica* haplotypes from Saudi Arabia, Figure 6. This analysis identified a *scutellata* haplotype (A4) sequence (FJ477987) as a potential shared ancestry from which all other groups deviated, Figure 6.

## 4 Discussion

Based on 24 morphometrical characteristics, discriminant analysis sharply distinguished *jemenitica* samples of Saudi Arabia from other neighboring subspecies such as *litorea*, *lamarckii*, and *syriaca* (Alattal et al., 2014a). All samples studied from Saudi Arabia (n = 179) exhibited the P0 elements in their COI-COII intergenic region (Alattal et al., 2014a), which makes them genuine members of the African lineage (Alburaki et al., 2011). Similar findings were recorded for *jemenitica* samples (n = 16) from the isolated Island of Socotra (Alattal et al., 2019). In the light of our COI-COII results, all analyzed samples (n = 15) exhibited the P0 element and thus belong to the African lineage (A), blasting with *jemenitica* and *syriaca* reference sequences, Table 3. The structural differences in the sequence following the tRNA_Leu_ gene have facilitated lineage and haplotype differentiation in *A. mellifera*. Specifically, samples from the (C) lineage and its haplotypes lack the P fragment, whereas the (A) lineage includes the P0 fragment (68 bp), and the (M) lineage contains the 54 bp P fragment (Cornuet et al., 1991; Garnery et al., 1993; Franck et al., 2000; Garnery et al., 1998a; Garnery et al., 1995; Rortais et al., 2011; Alburaki, 2011). The intertwining between *jemenitica* and *syriaca* observed in this study is yet to be understood by further investigations. Recent mtDNA sequencing studies have pointed out this genetic relationship between *jemenitica, syriaca,* and *lamarckii* (Boardman et al., 2020; Alghamdi and Alattal, 2020). Phylogenetic analysis of the cyt *b* offers different nuances. Most samples clustered within the African clade of *lamarckii* separated with a weak bootstrap value of 36 from the *syriaca* sub-lineage “Z” (Alburaki et al., 2013). This result indicates an undoubted genetic relationship with the African subspecies *syriaca* but still closer proximity with the *lamarckii* subspecies of Egypt, Figure 2. Only three Ethiopian and two Yemeni samples deviated from this cluster, showing closer relationships with *simensis* and *scutellata,* Figure 2. Notably, three of these five samples (Eth4, Eth5, Yem4) carried a significant deletion of 18 bp in their COI-COII P0 element, Table 3. Previous investigations on three *Apis m. jemenitica* samples from Ethiopia identified similar deletions in the P0 element (Alattal et al., 2019; Franck et al., 2001). It is safe to conclude that these deviating samples, at least for the Ethiopian samples (Eth4, 5, 6), are not *jemenitica* but *simensis*. This conclusion is supported by a 100% blast of their cyt *b* sequences with *simensis* references, Table 1. In contrast, the three other Ethiopian samples (Eth1, 2, 3) represent *jemenitica*, Table 1; Figure 2. Yemeni samples (Yem4, 8) showed closer similarity with *unicolor* and *simensis* than *jemenitica*, Table 1; Figure 2. From a biogeographical perspective, such phenomena are often observed in zones of subspecies interactions. The three major neighboring subspecies of *jemenitica* across the Red Sea in East Africa are *simensis*, *monticola,* and *litorea* (Ruttner, 1988). Unfortunately, very few sequences of the two latter subspecies were available to shed more light on the overlapping or interaction of these African subspecies with *jemenitica*. Even when available, most sequences were partial and could not be retained for analysis.

The COI gene offers a similar outcome, with the three most distant samples (Yem4, Eth4, 5) from the *jemenitica* cluster, a cluster that was firmly (Bootstrap = 97) separated from subspecies of all other lineages (C, M, and O), Figure 3. Nonetheless, at sequence blasting level, the COI gene seems to provide weak discriminative power as sequences fully blasted with multiple subspecies, Table 2. However, this may have occurred due to the relatively short length (218 bp) of our sequences in the COI gene. The COI phylogeny seems to fully agree with the cyt *b*, clustering the same four samples (Eth4, 5, Yem2, 4) as the most distant sequences from the rest, even at the intra-sample level, Figure 4. Interestingly, this observation was recorded to a great extend (Eth4, 5, Yem4) for the COI-COII intergenic region, Figure 4B. The COI-COII analysis showed a clear separation of four major clades: 1- M lineage, the African lineage (A) subdivided to 2- *jemenitica* (Group 1) which groups the majority of our samples with reference samples of *jemenitica* and *syriaca* haplotypes, 3- *jemenitica* (Group 2), which includes the far distinct samples (Yem4, Eht5, 4) clustering with haplotypes described to be *jemenitica* but all sampled from Ethiopia and finally 4-North African haplotypes, mainly characterized by *intermissa*, Figure 4A. The wide range of genetic diversity recorded for *jemenitica* populations is indeed unique, even at intra-sample level, Figure 4B. This might not be all surprising for the African lineage which was described to hold the highest genetic diversity among all lineages (Alburaki et al., 2011; Henriques et al., 2022; Franck et al., 2001). Nevertheless, a COI-COII haplotype diversity (*H*) of 0.980 in a population of 15 samples is unprecedented. The closest (*H*) that can be reported from previous investigations of African subspecies are 0.908 (Algeria, n = 42) as well as 0.701 (Algeria, n = 582) within *intermissa* populations (Achou et al., 2015; Ayad et al., 2024), 0.732 (Syria and Lebanon, n = 1,801) within *syriaca* populations (Alburaki et al., 2011). In contrast, mediocre haplotype diversity (*H =* 0.597, n = 1,063) was recorded for the honey bee populations of the United States, in which samples overwhelmingly belonged to the North Mediterranean lineage C (Alburaki et al., 2023). While all *intermissa* and *syriaca* studies cited above identified some degree of foreign mtDNA C-lineage introgression, our results showed no introgression of any kind to be recorded within the *jemenitica* populations of the Arabian Peninsula. The absence of introgression was also reported in previous *jemenitica* investigations from Socotra Island (Yemen) and Saudi Arabia (Alattal et al., 2019; Alattal et al., 2014b). Moreover, the studied populations of *jemenitica* accepted Tajima’s assumption of neutral evolution (D = - 1.25, *p* = 0.2), which suggests that these populations are not subjected to systematic human selection, Figure 5. This finding reflects what is observed in this region as most beekeepers practice traditional beekeeping with overwhelmingly no queen importations (Al-Ghamdi and Nuru, 2013b). The haplotype networks are in complete agreement with the COI-COII phylogenetic analysis. This intergenic region indicates that Ethiopian samples (Eth1,4,5) are genetically distant from those of Yemen, Saudi Arabia, Oman, and Jordan, Figure 5. When analyzed with available references, four clusters could be distinguished, Figure 6. These clusters represent two evolutionary lineages (A and M), with a significant intra-A lineage diversity. Two Ethiopian and one Yemeni sample clustered independently with previous reference samples from Ethiopia originating from a central *scutellata* haplotype (A4). North African *intermissa* haplotypes (A4p, A9j, A9l) clustered in closer proximity (SNP <4) to the central *scutellata* (A4) haplotype, Figure 6. The third clade relevant to this study comprises most of the analyzed samples clustering with *jemenitica* and *syriaca* reference haplotypes. This cluster suggests that *jemenitica* populations diverged from the *syriaca* haplotype (Z8), confirming our previous cyt *b* finding of intertwining between these two African subspecies despite being geographically distant. To understand this dual relationship, further investigations are needed with a sizable sampling from the contact zone between *jemenitica* (North) and *syriaca* (South).

In conclusion, this study provides new insights into the genetic diversity of the Arabian Peninsula’s honey bee populations and two neighboring countries. Despite the limited sample size, our data reveal significant genetic diversity within *jemenitica* populations. We identified and named eight novel COI-COII haplotypes from Yemen, Saudi Arabia, and Ethiopia that have never been reported. Evidence of subspecies overlapping was recorded, particularly between *jemenitica* and *syriaca*. Like many other autochthon subspecies, *jemenitica* is a remarkable honey bee subspecies that extends on a large geographical area and exemplifies adaptability and resilience in harsh environments. Its ecological importance and roles in both traditional and modern beekeeping underscore the need for continued research and conservation efforts to ensure its survival and support ecosystem health. Finally, our study was based on a limited sample size and focused solely on mtDNA markers. Future research should incorporate genomic DNA analysis with a significantly larger sample size to gain a more comprehensive understanding of the genetic diversity of *jemenitica* populations.

## Data Availability

The data presented in the study are deposited in the NCBI GenBank repository, accession numbers PQ460982 to PQ461055.
