# Supplementary material for: Genetic diversity and novel haplotypes of Apis mellifera jemenitica on the Arabian Peninsula: insights from mtDNA markers
Source: Front Genet. 2025 Apr 25;16:1532988. doi: 10.3389/fgene.2025.1532988 (PMC12061672; doi:10.3389/fgene.2025.1532988)
Supplement: Supplementary file 1 [file DataSheet1.pdf]

Table S1: NCBI reference sequences of three mtDNA loci used in this study to conduct phylogenetic analyses.

N	Gene	NCBI Accession	Listed species or subspecies	Known mtDNA lineage	Haplotype
1	Cyt b	FJ229480	*Apis cerana*	-	-
2	Cyt b	MT572360	*Apis m. adansonii*	A	-
3	Cyt b	MT572352	*Apis m. anatoliaca*	C/O	-
4	Cyt b	EF184047	*Apis m. carnica*	C	-
5	Cyt b	EF184048	*Apis m. carnica*	C	-
6	Cyt b	MT572351	*Apis m. carnica*	C	-
7	Cyt b	EF184050	*Apis m. caucasica*	C/O	-
8	Cyt b	EF184051	*Apis m. caucasica*	C/O	-
9	Cyt b	EF184052	*Apis m. caucasica*	C/O	-
10	Cyt b	EF184054	*Apis m. cypria*	C	-
11	Cyt b	EF184020	*Apis m. intermissa*	A	-
12	Cyt b	EF184021	*Apis m. intermissa*	A	-
13	Cyt b	EF184022	*Apis m. intermissa*	A	-
14	Cyt b	MT572347	*Apis m. lamarckii*	A	-
15	Cyt b	EF184043	*Apis m. ligustica*	C	-
16	Cyt b	MT572354	*Apis m. ligustica*	C	-
17	Cyt b	MT572350	*Apis m. meda*	C/O	-
18	Cyt b	MT572359	*Apis m. monticola*	A	-
19	Cyt b	MT572357	*Apis m. rutneri*	A	-
20	Cyt b	MT572353	*Apis m. sahariensis*	A	-
21	Cyt b	EF184027	*Apis m. scutellata*	A	-
22	Cyt b	EF184028	*Apis m. scutellata*	A	-
23	Cyt b	MT572358	*Apis m. scutellata*	A	-
24	Cyt b	MT572355	*Apis m. simensis*	A	-
25	Cyt b	EF184033	*Apis m. iberiensis*	M	-
26	Cyt b	MT572349	*Apis m. iberiensis*	M	-
27	Cyt b	EF184062	*Apis m. lamarckii*	A	-
28	Cyt b	EF184063	*Apis m. lamarckii*	A	-
29	Cyt b	MT572356	*Apis m. litorea*	A	-
30	Cyt b	EF184055	*Apis m. mellifera*	M	-
31	Cyt b	MT572348	*Apis m. mellifera*	M	-
32	Cyt b	EF184060	*Apis m. syriaca*	A	-
33	Cyt b	EF184061	*Apis m. syriaca*	A	-
34	Cyt b	MT572346	*Apis m. syriaca*	A	-
35	COI	OQ248419	*Apis m. jemenitica*	A	-
36	COI	AY114452	*Apis m. ligustica*	C	-
37	COI	AY114454	*Apis m. ligustica*	C	-
38	COI	AY114461	*Apis m. carnica*	C	-

39	COI	AY114456	*Apis m. ligustica*	C	-
40	COI	AY114479	*Apis m. iberiensis*	M	-
41	COI	AY114478	*Apis m. iberiensis*	M	-
42	COI	GU979501	*Apis m. ligustica*	C	-
43	COI	GU979500	*Apis m. caucasica*	C/O	-
45	COI	GU979499	*Apis m. carnica*	C	-
46	COI	MF100920	*Apis m. carpatica*	C	-
47	COI	MF100911	*Apis m. carpatica*	C	-
48	COI	MF100910	*Apis m. carpatica*	C	-
49	COI	AY114469	*Apis m. anatoliaca*	C/O	-
50	COI	AY114471	*Apis m. anatoliaca*	C/O	-
51	COI	AY114473	*Apis m. macedonica*	C	-
52	COI	AY114474	*Apis m. macedonica*	C	-
53	COI	AY114465	Buckfast Hybrid	C	-
54	COI	AY114476	*Apis m. adami*	A	-
55	COI	AY114477	*Apis m. adami*	A	-
56	COI	AY114463	*Apis m. carnica*	C	-
57	COI	AY114464	*Apis m. carnica*	C	-
58	COI	AY114466	*Apis m. caucasica*	C/O	-
59	COI	AY114467	*Apis m. caucasica*	C/O	-
60	COI-COII	OM219614	*Apis m. mellifera*	M	M1-825-5-USA
61	COI-COII	OM219616	*Apis m. mellifera*	M	M1-825-5-USA
62	COI-COII	KX463897	*Apis m. iberiensis*	M	M4p
63	COI-COII	KX463898	*Apis m. iberiensis*	M	M17i
64	COI-COII	MF428428	*Apis m. mellifera*	M	M8
65	COI-COII	KF274636	*Apis m. mellifera*	M	M4m
66	COI-COII	KF274634	*Apis m. mellifera*	M	M4k
67	COI-COII	KC149985	*Apis m. jemenitica*	A	KSA45
68	COI-COII	KC149983	*Apis m. jemenitica*	A	KSA4e
69	COI-COII	KC149988	*Apis m. jemenitica*	A	KSA6c
70	COI-COII	KC149987	*Apis m. jemenitica*	A	KSA6b
71	COI-COII	KC149984	*Apis m. jemenitica*	A	KSA4f
72	COI-COII	KC149989	*Apis m. jemenitica*	A	KSA6d
73	COI-COII	OM219608	African	A	A1-837-6-USA
74	COI-COII	OM219608	African	A	A1-837-6-USA
75	COI-COII	OM219611	African	A	A1-829-4-USA
76	COI-COII	OM994512	*Apis m. intermissa*	A	A12-828-3-DZA
77	COI-COII	OM994513	*Apis m. intermissa*	A	A6-828-2-DZA
78	COI-COII	OM994520	*Apis m. intermissa*	A	A9-828-2-DZA
79	COI-COII	OM994521	*Apis m. intermissa*	A	A9-828-2-DZA
80	COI-COII	OM107831	African	A	A4
81	COI-COII	OM107832	African	A	A4

82	COI-COII	OM219613	African	A	A3-830-4-USA
83	COI-COII	MW677211	African	A	A4p
84	COI-COII	OM107933	African	A	A4p
85	COI-COII	OM107934	African	A	A4p
86	COI-COII	OM107935	African	A	A4p
87	COI-COII	OM107936	African	A	A4p
88	COI-COII	OM107938	African	A	A4p
89	COI-COII	OM107937	African	A	A4p
90	COI-COII	FJ477987	*Apis m. scutellata*	A	A4
91	COI-COII	KX463840	*Apis m. intermissa*	A	A9q
92	COI-COII	KX463833	*Apis m. intermissa*	A	A9j
93	COI-COII	KX463835	*Apis m. intermissa*	A	A9l
94	COI-COII	FJ477999	*Apis m. jemenitica*	A	Y1b
95	COI-COII	FJ477998	*Apis m. jemenitica*	A	Y1a
96	COI-COII	FJ478002	*Apis m. jemenitica*	A	Y1c
97	COI-COII	FJ478000	*Apis m. jemenitica*	A	Y2a
98	COI-COII	FJ478003	*Apis m. jemenitica*	A	Y1d
99	COI-COII	FJ478001	*Apis m. jemenitica*	A	Y1b
100	COI-COII	KC149981	*Apis m. jemenitica*	A	KSA4c
101	COI-COII	HM236206	*Apis m. syriaca*	A	Z2
102	COI-COII	HM236210	*Apis m. syriaca*	A	Z8
103	COI-COII	KC149986	*Apis m. jemenitica*	A	KSA6a
104	COI-COII	KC149979	*Apis m. jemenitica*	A	KSA4a
105	COI-COII	KC149980	*Apis m. jemenitica*	A	KSA4b
106	COI-COII	KC149982	*Apis m. jemenitica*	A	KSA4d
